# Identification of biomarkers and exploration of mechanisms for atopic dermatitis based on transcriptome and scRNA-seq data analysis

**DOI:** 10.1097/MD.0000000000042291

**Published:** 2025-07-04

**Authors:** Yang Li, Xiao-Yong Ouyang, Wei-Bo Wen

**Affiliations:** a Traditional Chinese Medicine Department, Yunnan Maternal and Child Health Hospital, Kunming, China; b Department of Dermatology, Yunnan Provincial Traditional Chinese Medicine Hospital, Kunming, China; c Yunnan University of Traditional Chinese Medicine, Kunming, China.

**Keywords:** atopic dermatitis, bioinformatics, biomarkers, mechanism of AD, scRNA-seq, transcriptome

## Abstract

Atopic dermatitis (AD) is one of the most common chronic inflammatory skin diseases with complex pathogenesis and no effective treatment. This study aims to use bioinformatics methods to identify biomarkers and explore the mechanism for AD. We performed differential expression analysis based on transcriptome datasets GSE16161 and GSE32924. Next, the differentially expressed genes (DEGs) were subjected to Kyoto Encyclopedia of Genes and Genomes enrichment analysis. After integrating PPI network obtained from STRING database, we screened modular genes and identified candidate key genes by the MCC and degree algorithms. We selected genes with strong ROC performance and consistent expression levels as key genes, and constructed a nomogram to assess their potential as AD biomarkers. Finally, by analyzing the scRNA-seq dataset GSE180885, we identified the key cells associated with the key genes and conducted pseudotime analysis based on these key cells to explore the pathogenic mechanisms of AD. The results showed that 618 DEGs were identified and some important pathways, including Cytokine-Cytokine Receptor Interaction, Cell Cycle, Cell Adhesion Molecules and Calcium Signaling Pathway were screened out. Seven key genes were identified and they were *CCNA2*, *CCNB1*, *KIF2C*, *CEP55*, *MELK*, *CDC20*, and *CCNB2*. The nomogram analysis suggested that these key genes had the potential to serve as biomarkers for AD. Through scRNA-seq data analysis, we identified 9 cell subpopulations, with keratinocytes were identified as the key cells, and 6 out of 7 key genes showed significant expression in keratinocytes. Pseudotime analysis revealed that DEGs in keratinocytes played a vital role in the cellular differentiation process of AD. We successfully identified *CCNA2*, *CCNB1*, *KIF2C*, *CEP55*, *MELK*, *CDC20*, and *CCNB2*, as potential biomarkers for AD through transcriptomic and scRNA-seq data analysis.

## 1. Introduction

Atopic dermatitis (AD) is an itchy, inflammatory skin disease that often begins in infancy and has a chronic, relapsing course.^[1,2]^ Patients with AD experience symptoms such as itching, pain, and sleep difficulties, which significantly impact their lives.^[3]^ The ongoing treatment of AD also imposes a considerable economic burden on patients.^[4]^ The exact mechanisms of AD are not fully understood but may involve genetic factors, immune dysfunction, skin barrier damage, and local microbial infections.^[5,6]^ Environmental factors, such as seasonal changes, temperature fluctuations, and exposure to allergens like pollen, alcohol, and perfumes, can also trigger or exacerbate AD.^[7]^ Some studies have reported that the prevalence rate of AD ranges from 15% to 30% in children, while in adults, it ranges from 2% to 10%.^[8]^ Biomarker identification for AD significantly contributes to improved diagnostic accuracy, better monitoring disease progression, accurate assessment of treatment efficacy, and enhanced treatment outcomes.^[9,10]^

Current research into AD biomarkers has made significant strides in genetics, immunology, and mitochondrial function. Genetic studies have uncovered associations between specific gene variants and AD susceptibility, and immunological and inflammatory biomarkers have become essential tools for diagnosis and disease assessment, additionally the discovery of mitochondrial dysfunction has further elucidated the complex mechanisms underlying AD.^[11]^ Although these studies had opened new avenues for understanding the mechanism of AD, significant challenges in this field persist. Treating moderate-to-severe AD remains particularly challenging, with the inability to achieve a definitive cure and the recurrent nature of the disease severely impacting the health of AD patients.^[12]^

Some studies have identified various biomarkers for treating AD. However, the mechanisms of these biomarkers remain incompletely understood, as do the interactions among different biomarkers and their relationships with environmental factors.^[13]^ These issues often affect the accuracy of AD diagnosis and the precision of therapeutic strategies. Therefore, current research urgently needs to explore the specific roles of key biomarkers in AD. This will help improve the accuracy of AD diagnosis, enable more precise treatment strategies, and facilitate personalized therapy.

In this study, we employed bioinformatics methods such as differential expression analysis, protein–protein interaction (PPI) network construction, MCC, degree algorithms, nomogram analysis, receiver operating characteristic (ROC) analysis, and scRNA-seq data analysis to reveal how biomarkers influence the pathological process and mechanisms of AD at the level of biomarkers, biological functions, and pathways. These findings will aid in identifying new biomarkers for AD and provide new insights for the diagnosis and treatment of AD.

## 2. Materials and methods

### 2.1. Data source

Using the GEO database (https://www.ncbi.nlm.nih.gov/geo/),^[14]^ 4 transcriptome datasets of AD patients were obtained, including 2 training sets: GSE16161 and GSE32924, 1 validation set: GSE107361, and a scRNA-seq dataset: GSE180885.

GSE16161 dataset (microarray) contains 18 skin samples from 9 AD patients and 9 healthy controls. GSE32924 dataset (microarray) contains 33 skin samples from 25 AD patients and 8 healthy controls. GSE107361 dataset (microarray) contains 108 skin samples from 79 AD patients and 29 healthy controls. The scRNA-seq dataset GSE180885 contains 7 skin samples from 3 AD patients and 4 healthy controls.

### 2.2. Date preprocessing

To address batch effects and outliers in the dataset, the R package “Limma”^[15]^ was used to normalize the acquired microarray data by quartiles. As shown in Figure S1, Supplemental Digital Content, https://links.lww.com/MD/O868, a quality control box plot was drawn to check for the presence of outliers. Subsequently, log2 transformation was performed to obtain the gene expression matrix.

### 2.3. Differential expression analysis and KEGG pathway enrichment

To detect significant differentially expressed genes (DEGs) between the AD and the control group, we utilized the “Limma (v3.60.4)” package in R (v4.4.1) to identify the DEGs within the GSE16161 and GSE32924 datasets. The threshold was set at |log2FC| > 1, adjusted *P*-value (adj. p) < .05 and false discovery rate < 0.05.^[16,17]^ Take the intersection of up- and downregulated genes between GSE16161 and GSE32924 datasets, respectively.

We performed Kyoto Encyclopedia of Genes and Genomes (KEGG) pathway enrichment analysis on DEGs using R package “ClusterProfiler (v4.12.3)” to further identify specific biological pathways related to AD. Using the R package “ggplot2 (v3.5.1)” to draw a bubble plot, top 20 enriched KEGG pathways (based on GeneRatio) were shown, where GeneRatio represented the proportion of genes used for enrichment analysis among all genes within a specific pathway.

### 2.4. Construction of the PPI network and key gene identification

To reveal the PPI relationships among DEGs, we first utilized the STRING database (https://string-db.org, v12.0) to construct a PPI network, which was then imported into Cytoscape software (v3.7.1). The Molecular Complex Detection (MCODE) plugin was employed to perform module analysis and identify module genes. Subsequently, we used the cytoHubba plugin to score and rank nodes according to network characteristics,^[18]^ including maximal clique centrality (MCC) and degree algorithm.^[19]^ The intersection genes of these 2 top 10 gene lists (MCC top 10 and degree top 10) were taken as the candidate genes. To evaluate the ability of these candidate genes to distinguish atopic AD samples from normal samples, ROC curves were plotted based on the dataset GSE32924 and validated using the validation set GSE107361. At the same time, the gene expression levels of candidate genes in datasets GSE32924 and GSE107361 were analyzed (*P* < .05). The candidate genes with the same expression trend and passed the ROC validation were used as key genes.

### 2.5. Nomogram

To construct a predictive nomogram, the R package ‘rms (v6.8.1)’ was employed to generate the nomogram. Each gene’s expression value corresponded to a score on the nomogram. The total score was the sum of individual gene scores. ROC analysis and decision curve analysis (DCA) were then conducted to evaluate the prediction ability of the nomogram model.

### 2.6. scRNA-seq data processing

For the scRNA-seq dataset GSE180885, we used the “CreateSeuratObject” function from the R package “Seurat (v5.1.0)”^[20]^ to perform data filtering, retaining genes expressed in at least 3 cells and cells that have detected more than 200 genes (min.cells = 3, min.features = 200). Using the “Seurat” package’s “PercentageFeatureSet” function, we calculated mitochondrial genes and retained cells with a mitochondrial gene proportion of <10%.

We used the “NormalizeData” function from the “Seurat” package to standardize the data and the “FindVariableFeatures” function to identify highly variable genes among cells. Subsequently, we performed principal component analysis for dimensionality reduction and ultimately selected 30 principal components for subsequent clustering analysis.

### 2.7. Cell clustering

Unsupervised clustering analysis of the cells was performed using the “FindNeighbors” and “FindClusters” functions, resulting in 20 clusters at the default resolution of 0.5. The clustering results were visualized using uniform manifold approximation and projection (UMAP). Based on the previous research,^[21]^ we obtained cell markers for various cell types and annotated each cluster with its corresponding cell type, visualized the results using UMAP. To validate the functions of the key genes, we used the “FindMarkers” function to perform differential expression analysis of these key genes across different cell types. Meanwhile, the differences in the expression of key genes across all cell types were examined, and the cell type with the highest number of significant differences (*P* < .05) in key genes was used as the key cell, and displayed the results using a dot plot.

### 2.8. Key cell pseudotime trajectory analysis

We extracted the data corresponding to the key cells, keratinocytes, and used the “differentialGeneTest” function from the R package “Seurat” to identify DEGs within these key cells. Subsequently, we performed pseudotime analysis using the R package “monocle (v2.32.0)”^[22]^ to plot trajectory maps of AD cells and NHS (normal human skin) cells, as well as a trajectory map of all cells (combining AD cells and NHS cells together).

### 2.9. Statistical analysis

R software (v4.3.1) was utilized for statistical analysis. The differences between groups were assessed using either the t-test or the Wilcoxon rank-sum test, depending on the data distribution. *P* < .05 was considered statistically significant.

## 3. Results

### 3.1. Differential expression analysis

**Figure 1.**
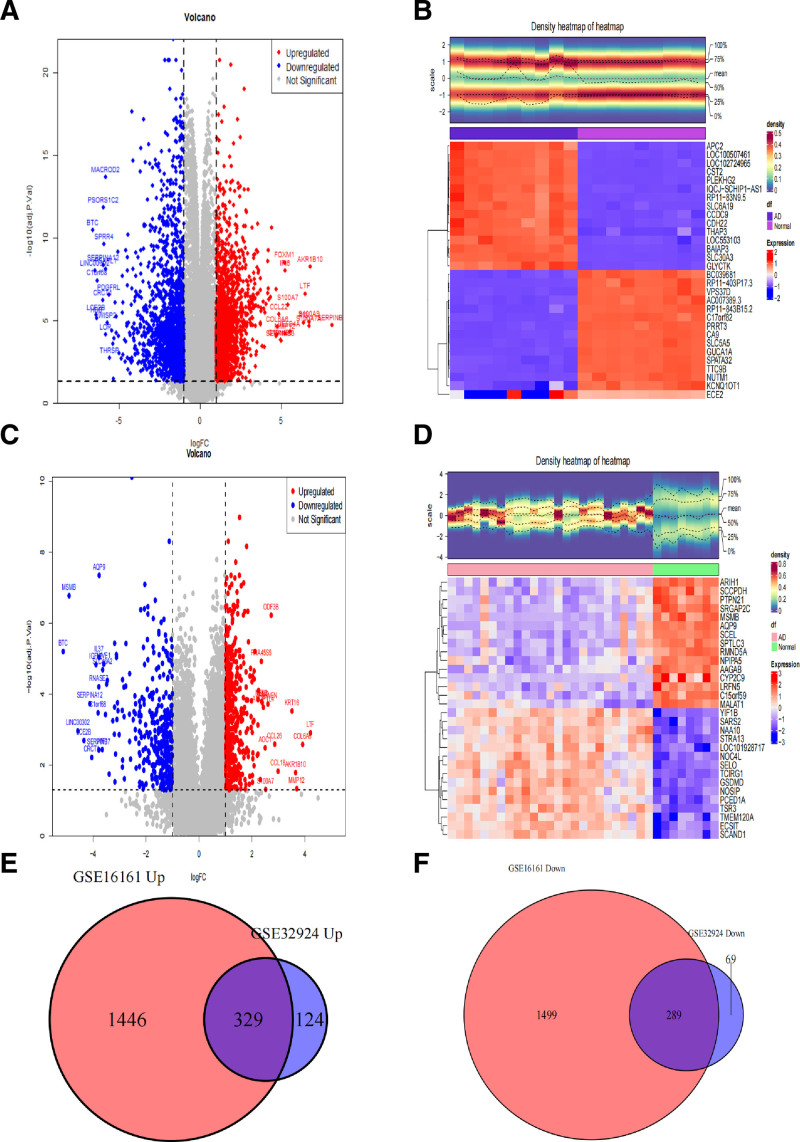
Differential expression analysis. (A) Volcano plot of differential expression analysis between disease samples and normal samples in dataset GSE16161, showing the top 15 upregulated and downregulated genes. Upregulated genes are represented in red, and downregulated genes are represented in blue. (B) Heatmap of differential expression analysis in dataset GSE16161, with redder colors indicating higher expression levels and bluer colors indicating lower expression levels. (C) Volcano plot of differential expression analysis between disease samples and normal samples in dataset GSE32924. (D) Heatmap of differential expression analysis in dataset GSE32924. (E) Venn diagram showing the intersection of upregulated genes between dataset GSE16161 and dataset GSE32924. (F) Venn diagram showing the intersection of downregulated genes between dataset GSE16161 and dataset GSE32924.

We performed differential expression analysis on dataset GSE16161 and dataset GSE32924. In dataset GSE16161, a total of 3563 DEGs were identified, among which 1775 genes were upregulated and 1788 genes were downregulated in the AD group (Fig. 1A). The top 15 upregulated and downregulated genes showed significant differences between the AD group and the control group (Fig. 1B), indicating that the results of the differential expression analysis are robust. In the differential gene analysis of dataset GSE32924, a total of 811 DEGs were identified, with 453 genes upregulated and 358 genes downregulated in the AD group (Fig. 1C). Similar to GSE16161, the top 15 upregulated and downregulated genes in dataset GSE32924 also showed significant differences in expression levels between the AD group and the control group (Fig. 1D). Dataset GSE16161 and dataset GSE32924 shared 329 commonly upregulated genes and 289 commonly downregulated genes (Fig. 1E and F).

### 3.2. KEGG pathway enrichment and key gene identification

**Figure 2.**
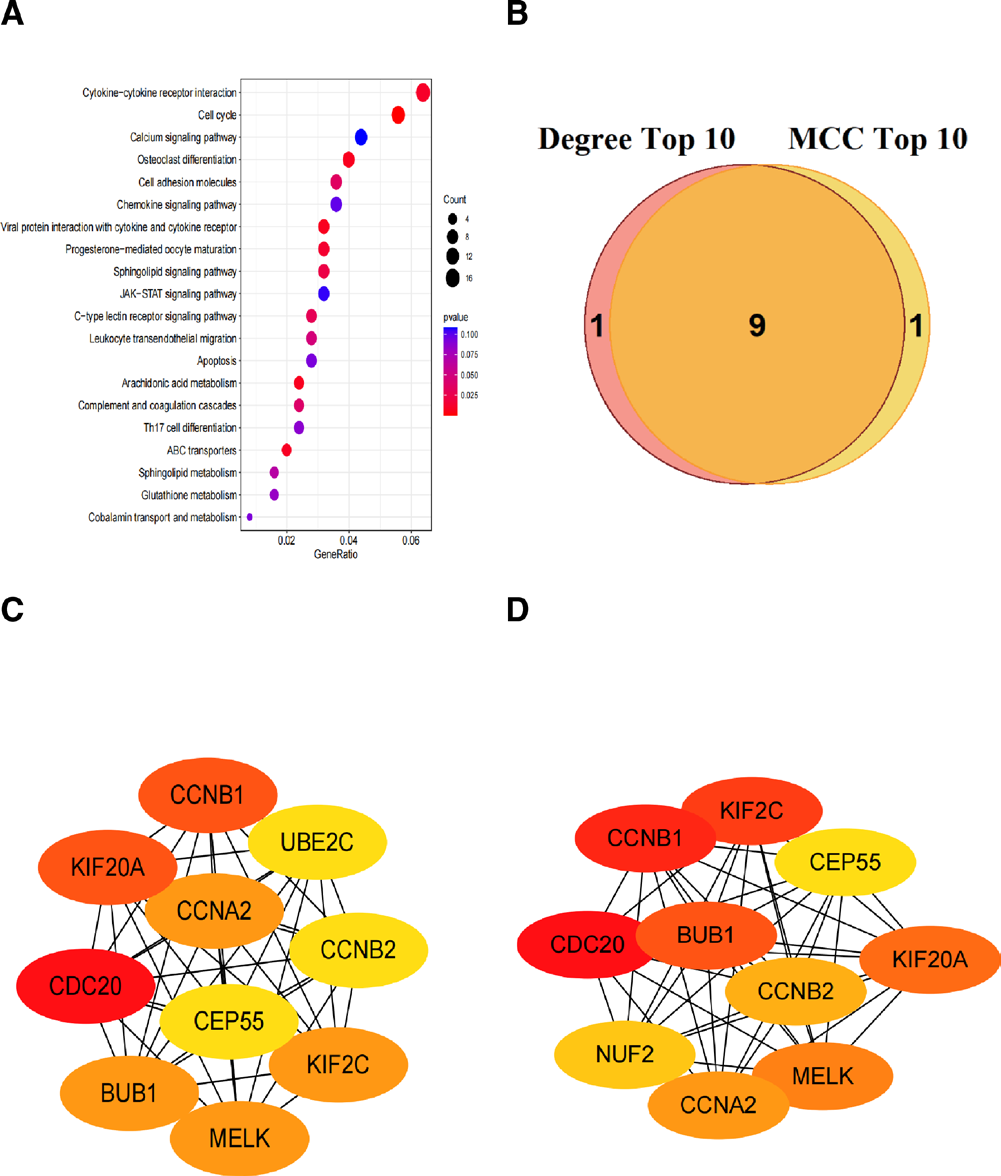
KEGG pathway enrichment and identification of key genes using PPI networks. (A) Bubble plot of KEGG pathway enrichment analysis. GeneRatio represents the proportion of genes used for enrichment analysis among all genes in a given pathway. The size of the circle indicates the number of genes used for enrichment analysis that appear in a given pathway. The color of the circle represents the *P*-value, with bluer colors indicating higher *P*-values and redder colors indicating lower *P*-values. The top 20 pathways are shown based on the GeneRatio. (B) Venn diagram illustrating the intersection of the top 10 Degree genes and the top 10 MCC genes. (C) PPI network of the top 10 degree genes, with colors ranging from yellow to red, where darker colors represent higher degree scores. (D) PPI network of the top 10 MCC genes, with colors ranging from yellow to red, where darker colors represent higher MCC scores. KEGG = Kyoto Encyclopedia of Genes and Genomes, MCC = maximal clique centrality, PPI = protein–protein interaction.

We combined the 329 commonly upregulated genes and 289 commonly downregulated genes shared by dataset GSE16161 and dataset GSE32924, resulting in a total of 618 DEGs. Using these 618 genes, we conducted KEGG pathway enrichment analysis, and the top 5 pathways were Cytokine-cytokine receptor interaction, Cell cycle, Calcium signaling pathway, Osteoclast differentiation, and Cell adhesion molecules (Fig. 2A). “Cytokine-cytokine receptor” interaction is a pathway related to the binding of cytokines and their receptors, playing a crucial role in the immune system. “Cell cycle” is a pathway associated with cell division. “Calcium signaling pathway” is related to calcium metabolism. “Osteoclast differentiation” is a pathway associated with osteoclast differentiation. “Cell adhesion molecules” is related to cell adhesion molecules. These pathways play important roles in some biological processes such as immunity, cell division, and calcium metabolism.

We imported 618 DEGs into the STRING database to construct a PPI network. PPI network was visualized using Cytoscape software, and module detection was performed using the MCODE plugin to screen out 16 genes. Subsequently, we used the cytoHubba plugin in Cytoscape to identify the top 10 genes based on the Degree algorithm. These genes were *CCNA2*, *CCNB1*, *CCNB2*, *CDC20*, *CEP55*, *KIF20A*, *KIF2C*, *BUB1*, *UBE2C*, and *MELK* (Fig. 2C). Additionally, we applied the MCC (Maximal Clique Centrality) algorithm to select the top 10 genes, including *CCNA2*, *CCNB1*, *CCNB2*, *CDC20*, *CEP55*, *KIF20A*, *KIF2C*, *BUB1*, *MELK*, and *NUF2* (Fig. 2D). Finally, by intersecting the Degree top 10 genes with the MCC top 10 genes, we identified 9 candidate key genes, including *CCNA2*, *CCNB1*, *CCNB2*, *CDC20*, *CEP55*, *KIF20A*, *KIF2C*, *BUB1*, and *MELK* (Fig. 2B).

**Figure 3.**
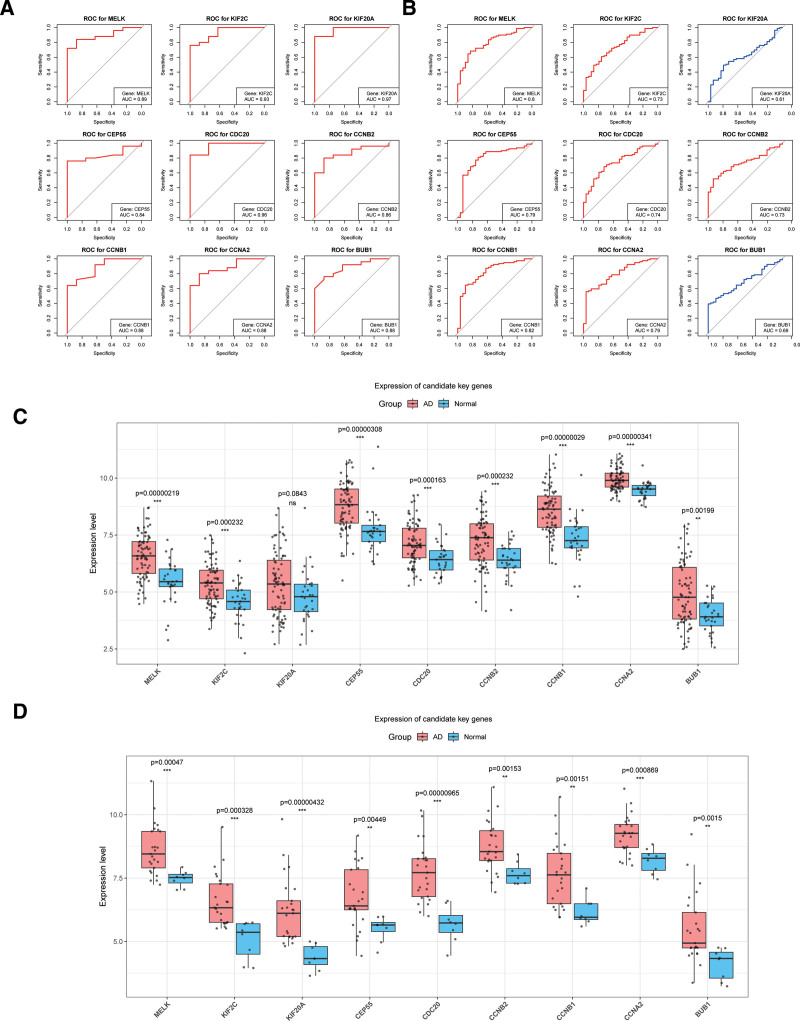
(A) ROC curves of the key genes in the GSE32924 dataset. (B) ROC curves of the key genes in the GSE107361 dataset. A larger AUC indicates a better performance of the gene in distinguishing disease samples from normal samples. (CA and D) Boxplots of expression levels of candidate key genes in dataset GSE32924 (C) and GSE107361 (D). AUC = area under the curve, ROC = receiver operating characteristic.

Next, we plotted ROC curves based on the expression values of these 9 candidate key genes in datasets GSE32924 and GSE107361. Among them, 7 genes had AUC values exceeding 0.7 in both the GSE32924 and GSE107361 dataset, confirming them as identified key genes (Fig. 3A and B). Expression analysis showed that the expression levels of candidate key genes in the training set GSE32924 and the validation set GSE107361 were significantly different between the AD and control groups, and all of them were significantly upregulated in the AD group (Fig. 3C and D). Ultimately, 7 key genes were identified, containing *CCNA2*, *CCNB1*, *KIF2C*, *CEP55*, *MELK*, *CDC20*, and *CCNB2*.

### 3.3. Nomogram

**Figure 4.**
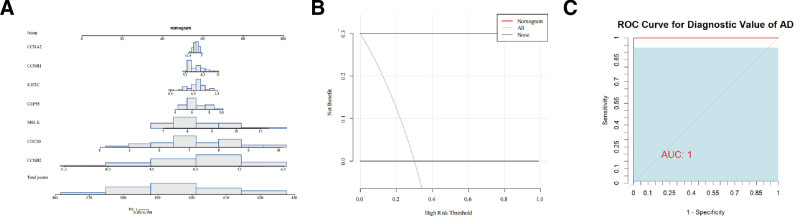
(A) Nomogram. The figure displays the relative scores for each gene, with higher scores indicating better ability to distinguish between disease samples and normal samples. (B) DCA curve. The “All” curve represents all samples being predicted as positive, the “None” curve represents all samples being predicted as negative, and the “Nomogram” curve represents the prediction results of the nomogram model. (C) ROC curve of the nomogram model. DCA = decision curve analysis, ROC = receiver operating characteristic.

We plotted a nomogram for the 7 key genes in the GSE32924 dataset. As shown in Figure 4, the scores for the expression values of all 7 genes are relatively high, with some samples corresponding to the genes MELK, CDC20, and CCNB2 scoring close to 100 (Fig. 4A). Furthermore, based on the nomogram model of these 7 genes, we also plotted the DCA curve (Fig. 4B) and ROC curve (Fig. 4C). The DCA curve is favorable, and the AUC value of the ROC curve equals 1, indicating the outstanding predictive performance of the nomogram model. These results suggested that the 7 key genes we identified could effectively distinguish disease samples from normal samples, making them potential biomarkers for AD.

### 3.4. Identification of key cell types based on scRNA-seq data analysis

**Figure 5.**
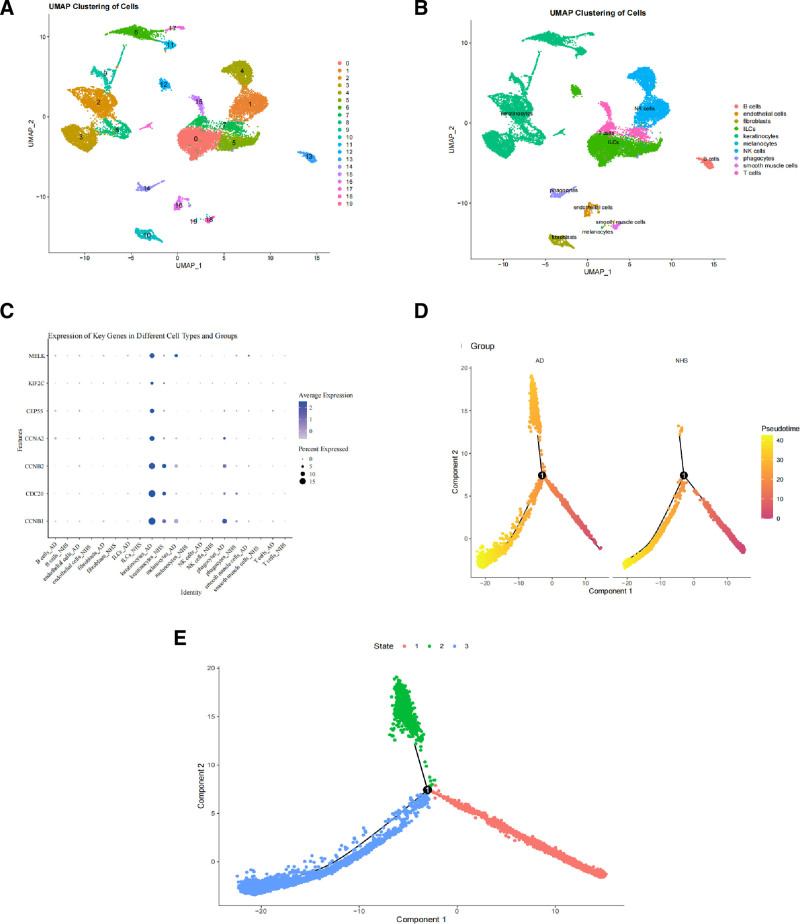
Identification of key cell types based on scRNA-seq data analysis. (A) UMAP for dimension reduction plot of cell clustering. (B) UMAP plot with cell type annotations. (C) Dot plot showing the expression of 7 key genes across different cell types. The darker the circle color, the higher the average expression value. The larger the circle size, the greater the proportion of cells expressing that gene. (D) Trajectory plot of AD cells and NHS (normal human skin) cells. (E) Trajectory plot of all cells. AD = Atopic dermatitis, UMAP = uniform manifold approximation and projection.

To explore the pathogenic mechanisms of AD at the single-cell level, we analyzed the scRNA-seq dataset GSE180885 and clustered the cells into 20 clusters (Fig. 5A). Subsequently, based on the cell markers from previous study,^[21]^ we annotated the 20 clusters to identify 10 cell types (Fig. 5B): KLRB1 + and IL7R + ILCs (innate lymphoid cells) were located in clusters 0, 5, and 13. The KLRD1 + and GNLY + NK (natural killer) cells were present in clusters 1, 4. CD3D + T cells were predominated in cluster 7. JCHAIN + and IGKC + B cells were present in cluster 12; the phagocytes marked by HLA-DQB1+, LYZ + and CD74 + were located in cluster 14. ACTA2 + and TAGLN + smooth muscle cells were found in cluster 18. PMEL+, MLANA + and KIT + melanocytes were predominated in cluster 19; RAMP2 + and PECAM1 + endothelial cells were located in cluster 16. COL1A2 + and COL6A2 + fibroblasts were present in cluster 10. KRT14 + keratinocytes were located in clusters 2, 3, 6, 8, 9, 11, and 17.

To investigate the functions of key genes in specific cell types, we performed differential expression analysis across different cell types. We discovered that 6 out of 7 key genes were significantly differentially expressed in keratinocytes (Fig. 5C and Table 1), thereby identifying keratinocytes as the key cell type. Subsequently, we identified the DEGs in keratinocytes and performed pseudotime analysis based on the expression values of these genes. As showed in trajectory plots, without distinguishing between AD cells and NHS (normal human skin) cells, all cells differentiated into 3 states (Fig. 5E). However, when AD cells and NHS cells were separated, an additional branch emerged specifically in AD cells (Fig. 5D), indicating that the identified key cell type, keratinocytes, plays a crucial role in the differentiation of AD cells.

**Table 1:** Differential expression analysis results of 6 key genes in keratinocytes.

Gene	*P*-value	Avg. log2FC	pct.1	pct.2	Adj. *P*-value
MELK6	4.89 × 10^−27^	1.488	0.112	0.028	1.00 × 10^−22^
CEP556	2.39 × 10^−24^	1.952	0.1	0.026	4.91 × 10^−20^
CCNA26	4.48 × 10^−24^	1.992	0.116	0.037	9.19 × 10^−20^
CCNB15	2.53 × 10^−20^	0.952	0.173	0.081	5.19 × 10^−16^
KIF2C6	1.18 × 10^−12^	1.221	0.065	0.022	2.41 × 10^−08^
CCNB26	5.06 × 10^−09^	−0.304	0.152	0.092	1.04 × 10^−04^

“pct.1” represents the percentage of cells in which the expression of the gene or feature is detected in ident.1. “pct.2” represents the percentage of cells in which the expression of the gene or feature is detected in ident.2. ident.1 represents the atopic dermatitis group, ident.2 represents the normal human skin group.

## 4. Discussion

Atopic dermatitis is a chronic, recurrent inflammatory skin disease, which is characterized by intense itching, symmetric distribution, and eczematous lesions. Its development is associated with many factors, such as genetic predisposition, environmental influences, immune abnormalities, and pharmacological mediators. In this study, we utilized various bioinformatics methods such as differential gene expression analysis, KEGG pathway enrichment analysis, PPI network analysis, MCC and Degree algorithms, ROC analysis, nomogram analysis, and scRNA-seq data analysis to identify key biomarkers affecting AD. These biomarkers can help clinicians better understand the disease state, develop personalized treatment plans, and monitor treatment outcomes.

Through differential expression analysis of transcriptomic datasets, we obtained 618 DEGs. KEGG pathway analysis of these DEGs revealed many significant pathways, such as Cell Cycle, Cytokine-Cytokine Receptor Interaction, Cell Adhesion Molecules and Calcium Signaling Pathway. Cell Cycle is the process by which a cell progresses from 1 division to the next, regulating cell growth and division.^[23]^ Cytokines are proteins that transmit signals between cells, regulating immune responses by binding to their receptors on cell membrane.^[24]^ Many cytokines and their receptors play key roles in activating and modulating inflammatory responses in the pathogenesis of AD. For example, cytokines *IL-4*, *IL-13*, and *IL-31* promote the recruitment and activation of inflammatory cells, thereby exacerbating skin inflammation.^[25]^ Some studies had shown that cell adhesion molecules, such as ICAM-1 and ICAM-3, are upregulated in the skin tissues of AD patients. This may be related to the homing of T-cell subsets to allergen-exposed skin, and the high expression of these molecules could be associated with immune cell migration and inflammatory responses.^[26]^ Calcium ions (Ca2+) and their concentration gradients in the epidermis are crucial for regulating some skin functions, such as keratinocyte differentiation, skin barrier formation, and permeability barrier homeostasis. In inflammatory skin conditions such as AD, impaired skin barrier function is a primary characteristic, and abnormalities in calcium signaling pathway may contribute to this dysfunction.^[27]^ These results indicated that the enriched pathways play an important role in the pathogenesis of AD.

The 7 key genes identified through MCODE, ROC curve analysis and validated in external independent verification set, they are: *CCNA2*, *CCNB1*, *KIF2C*, *CEP55*, *MELK*, *CDC20*, and *CCNB2*. *CCNA2* is a gene that functions during the G1/S and G2/M transitions by forming complexes with cyclin-dependent kinases such as CDK2, promoting cell cycle progression.^[28,29]^ A paper has shown that CCNA2 is involved in cell proliferation and epithelial-mesenchymal transition.^[30]^ Consistent with previous findings, *CCNA2* was significantly enriched in the Cytokine-Cytokine Receptor Interaction pathway, which plays a key role in AD genesis.^[31]^ Cytokine-Cytokine Receptor regulating inflammatory factors such as IL-19, CXCL13 and IL-14 to influence the inflammatory response, and influencing immune cell activation.^[32]^ In the pathogenesis of AD, CCNA2 may affect the proliferation and differentiation of skin cells, as well as participate in inflammatory responses, thereby influencing disease progression.

*CCNB1* gene encodes G2/mitotic-specific Cyclin B1, which plays a crucial role during the G2/M transition by forming a complex with CDK1.^[33]^ This complex promotes cell entry into mitosis through the phosphorylation of specific substrates.^[34]^ KIF2C gene encodes Kinesin family member 2C, which forms a complex with KIF18B and serves as a primary contributor to the microtubule plus-end depolymerase activity within mitotic cells. It regulates the switching of microtubules at the kinetochores and plays a key role in chromosome separation.^[35]^ Upregulation of KIF2C could promote tumor cell migration, invasion, chemoresistance, and inhibition of DNA damage repair. It is also highly associated with microRNAs, CD4 + T-cell, and CD8 + T-cell tumor immune infiltration.^[36]^ CEP55 gene encodes centrosomal protein 55 kDa that plays an important role during cell mitosis, particularly in late mitosis and cytokinesis.^[37]^ During cytokinesis, CEP55, in conjunction with PDCD6IP and TSG101, is recruited to the midbody, playing an essential role in the successful completion of cell division.^[38]^ Additionally, CEP55 is associated with the development of the brain and kidneys.^[39]^ CEP55 may enhance epithelial-mesenchymal transition through the PI3K/AKT/mTOR pathway, which plays a crucial role in tumor development and metastasis.^[40]^

*CDC20* (cell division cycle 20) is a cell cycle regulatory protein that forms an E3 ubiquitin ligase by binding to the Anaphase-Promoting Complex/Cyclosome (APC/C), and regulates cell mitosis by controlling substrate degradation.^[41]^ A further key gene, *CCNB2*, has been identified as a cell cycle protein that is primarily involved in the G2/M phase transition.^[42]^ A substantial body of research has established a strong association between *CCNB2* function and a variety of tumor prognosis and pathogenesis. Nevertheless, studies in AD remain limited.^[43–45]^ Comprehensive bioinformatics analysis and experiments conducted by Yin et al unearthed significantly elevated *CCNB2* expression levels in AD patients, which is consistent with our observations.^[46]^ *MELK*, a member of the serine/threonine family of proteins, has been demonstrated to play a role in various cellular processes, including cell proliferation, apoptosis and cell cycle progression.^[47,48]^ Apoptosis constitutes one of the pathogenic mechanisms underlying AD. Studies have revealed that skin-homing T cells, eosinophils and keratinocytes exhibit dysregulated apoptosis, contributing to both the onset and the persistence of AD.^[49]^ These findings suggest that key genes may play a critical role in the pathogenesis of AD by regulating pathways such as the cell cycle and cytokine-cytokine receptor interactions, which in turn affect the proliferation, differentiation, inflammatory response and immune response of skin cells. However, this requires further experimental validation.

Through scRNA-seq data analysis, we identified cellular heterogeneity in AD, revealing 10 distinct cell subpopulations, namely: innate lymphoid cells, natural killer cells, T cells, B cells, phagocytes, smooth muscle cells, melanocytes, endothelial cells, fibroblasts and keratinocytes, in agreement with the findings of Zhang et al.^[50]^ The role of these cells in AD has been widely reported, e.g. T cells and innate lymphoid cells are involved in the type 2 immune response leading to skin barrier dysfunction, which is one of the typical features of AD.^[51–53]^ In addition, macrophages, fibroblasts and endothelial cells have been shown to influence the inflammatory response, which is crucial for the onset and development of AD.^[54–56]^ Among the 7 key genes (CCNA2, CCNB1, KIF2C, CEP55, MELK, CDC20, and CCNB2), 6 exhibited significant differential expression in keratinocytes. Keratinocytes are the primary cells of the epidermis, exhibiting different morphologies and functions across various layers of the skin. These cells produce keratin, a tough fibrous protein that provides an effective protective barrier for the skin.^[57]^ Keratinocytes play a crucial role in maintaining skin health and repair. They recognize antigens through nucleotide-binding oligomerization domain-like receptors (NOD-like receptors) and toll-like receptors, and upon activation, the expression levels of cytokines, chemokines, and antimicrobial peptides are upregulated, helping to initiate the skin’s immune response.^[58]^ Keratinocytes express many inflammatory mediators, such as thymic stromal lymphopoietin (TSLP), tumor necrosis factor α (TNF-α), and some interleukins such as IL-1α, IL-1β, and IL-18, which mediate skin inflammation. Additionally, a lack of antimicrobial peptide expression due to keratinocyte damage may increase the skin’s susceptibility to viral, bacterial, and fungal infections.^[59]^ Therefore, keratinocytes are important for maintaining skin barrier and regulating the skin’s immune response. Changes in their state and function are directly linked to the onset and progression of skin diseases.

However, there are some limitations of our study. First, the data were obtained from public databases, and the sample size and the source somewhat limited the generalizability of our findings. Second, there is a lack of consideration of the corresponding clinical information, which limits our ability to fully explore the role of key genes in the pathogenesis, prognosis and treatment of AD. Third, the analysis of the single-cell transcriptome was slightly insufficient and lacked experimental validation. Finally, although we used external independent datasets to validate the expression levels and ROC of key genes, the findings were all obtained based on bioinformatics analyses, and further ex vivo and in vivo experimental validation is still lacking. Therefore, collecting clinical samples and exploring the role of key genes in keratinocytes, etc will be the primary focus of our future research. Nevertheless, our findings provide new references and insights into the pathogenesis of AD, which are of great significance.

## 5. Conclusions

Various bioinformatics methods such as differential gene expression analysis, KEGG pathway enrichment analysis, PPI network analysis, ROC analysis, nomogram analysis and scRNA-seq data analysis were used to identify biomarkers for diagnosing AD. The identified biomarkers are CCNA2, CCNB1, KIF2C, CEP55, MELK, CDC20, and CCNB2. These biomarkers influence the proliferation, differentiation, and inflammatory responses of skin cells, potentially contributing to the onset of AD. Through scRNA-seq data analysis, 6 out of the 7 key genes (CCNA2, CCNB1, KIF2C, CEP55, MELK, CDC20, CCNB2) showed significant differential expression in keratinocytes. Therefore, keratinocytes can serve as potential biomarkers for diagnosing AD.

## Acknowledgments

Sincerely thanks to all the people and organizations that contributed to this study.

## Author contributions

**Conceptualization:** Yang Li, Xiao-Yong Ouyang.

**Methodology:** Wei-Bo Wen.

**Software:** Yang Li.

**Validation:** Yang Li, Xiao-Yong Ouyang, Wei-Bo Wen.

**Writing – original draft:** Yang Li.

**Writing – review & editing:** Wei-Bo Wen.

## Supplementary Material


