# Supplementary figures and images for: Identification of biomarkers and exploration of mechanisms for atopic dermatitis based on transcriptome and scRNA-seq data analysis
Source: Medicine (Baltimore). 2025 Jul 4;104(27):e42291. doi: 10.1097/MD.0000000000042291 (PMC12237329; doi:10.1097/MD.0000000000042291)

Supplementary Figure S1 quality control box plot

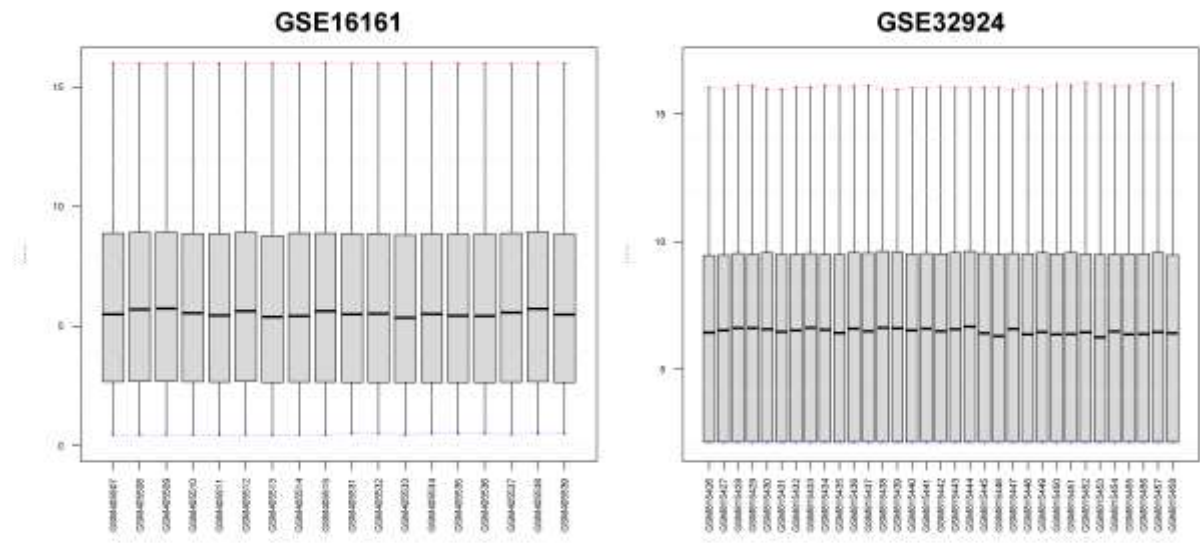

Supplement: Supplementary file 1 [file medi-104-e42291-s001.pdf]
